# Neutralizing Antibodies Against the SARS-CoV-2 Omicron Variant (BA.1) 1 to 18 Weeks After the Second and Third Doses of the BNT162b2 mRNA Vaccine

**DOI:** 10.1001/jamanetworkopen.2022.12073

**Published:** 2022-05-13

**Authors:** Ria Lassaunière, Charlotta Polacek, Anders Frische, Lasse Boding, Susanne Gjørup Sækmose, Morten Rasmussen, Anders Fomsgaard

**Affiliations:** 1 Department of Virus and Microbiological Special Diagnostics, Statens Serum Institut, Copenhagen, Denmark; 2 Danish National Biobank, Statens Serum Institut, Copenhagen, Denmark; 3 Department of Clinical Immunology, Zealand University Hospital, Naestved, Denmark

## Abstract

This cohort study examines neutralizing antibodies against the SARS-CoV-2 Omicron variant (BA.1) after the second and third doses of the BNT162b2 mRNA vaccine among Danish adults.

## Introduction

The SARS-CoV-2 Omicron variant of concern^1^ (VOC) is highly resistant to vaccine-induced antibody neutralization^2^ and associated with a decline in vaccine efficacy within the first 3 months following the primary 2-dose regimen of the SARS-CoV-2 BNT162b2 (Pfizer/BioNTech) mRNA vaccine^3,4^ and from 10 weeks after a third BNT162b2 dose.^4^ To associate Omicron-specific neutralizing antibody levels with reported vaccine efficacies, we performed a temporal analysis of virus neutralization responses against an ancestral strain (D614G), the Delta VOC, and the Omicron VOC (BA.1) following 2 or 3 doses of the BNT162b2 vaccine.

## Methods

This cohort study includes Danish adults who received 2 or 3 doses of BNT162b2 between January 2021 and October 2021 or were previously infected prior to February 2021 and then vaccinated. In the latter group, given the timeframe, individuals became infected with ancestral SARS-CoV-2 strains before VOCs became dominant in Denmark. We determined 50% serum neutralization titers using a live virus microneutralization assay^5^ (eAppendix in the Supplement). Paired and nonpaired numerical measurements were compared using the Wilcoxon matched-pairs signed rank test and Mann-Whitney *U* test, respectively. The association between continuous variables were assessed using the Spearman correlation analysis. This study constitutes national infectious disease surveillance performed on excess biological material by Statens Serum Institut, an institute under the Danish Ministry of Health, according to Section 222 of the Danish Health Act and following data protection regulations. This study is therefore exempt from ethical review and did not require patient consent. We followed the Strengthening the Reporting of Observational Studies in Epidemiology (STROBE) reporting guideline.

## Results

**Figure 1.**
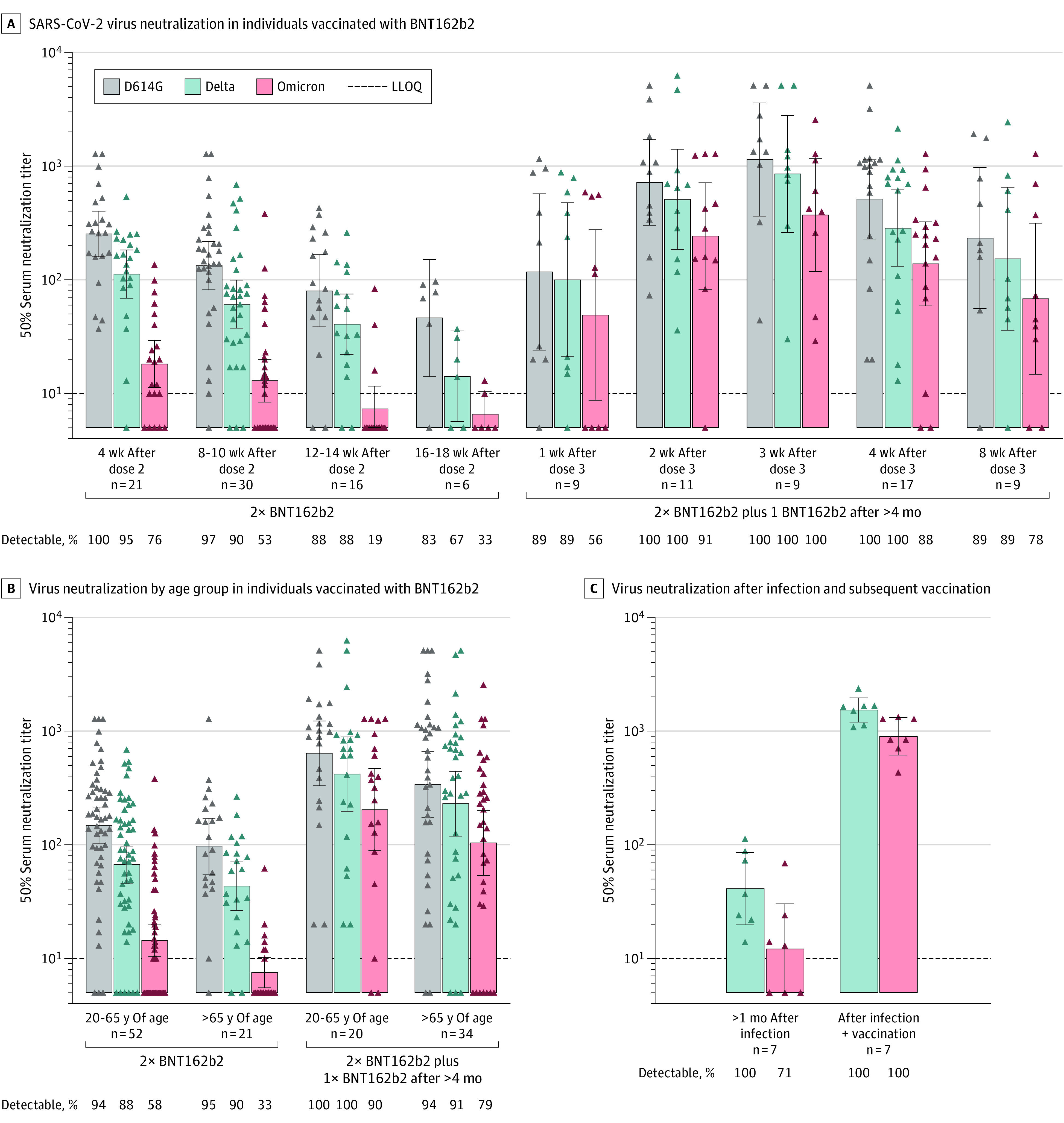
Temporal Virus Neutralizing Antibody Responses Against Ancestral SARS-CoV-2 Strain (D614G), Delta Variant (B.1.617.2), and Omicron Variant (B.1.1.529, BA.1) A, Live virus neutralization titers for a cross-sectional cohort of individuals vaccinated with BNT162b2 (Pfizer/BioNTech) vaccine (n = 128) at 4 to 18 weeks following the second dose in the primary 2-dose vaccination series and 1 to 8 weeks following a third BNT162b2 dose administered more than 4 months after the second dose. B, Live virus neutralization titers stratified by age group 4 to 18 weeks after the primary 2-dose BNT162b2 vaccination series and 1 to 8 weeks after the third BNT162b2 dose. C, In a longitudinal cohort of individuals (n = 7) who became infected before January 2021—before the Alpha and Delta variants became dominant in Denmark—virus neutralization titers were determined 46 to 186 days after a polymerase chain reaction positive test (median: 65 days) and after subsequent vaccination more than 6 months after the infection, primarily within 5 weeks postvaccination. The viral targets in the microneutralization assays were Danish clinical isolates passaged twice in Vero E6 cells and sequenced to confirm lineage-specific spike variations. Data points represent individual 50% serum neutralization titers. Bars represent the geometric mean titer (indicated above bar) and error bars the 95% CI. The lower limit of quantitation (LLOQ) was 10 and all values below the LLOQ were set to 0.5 times the LLOQ.

**Figure 2.**
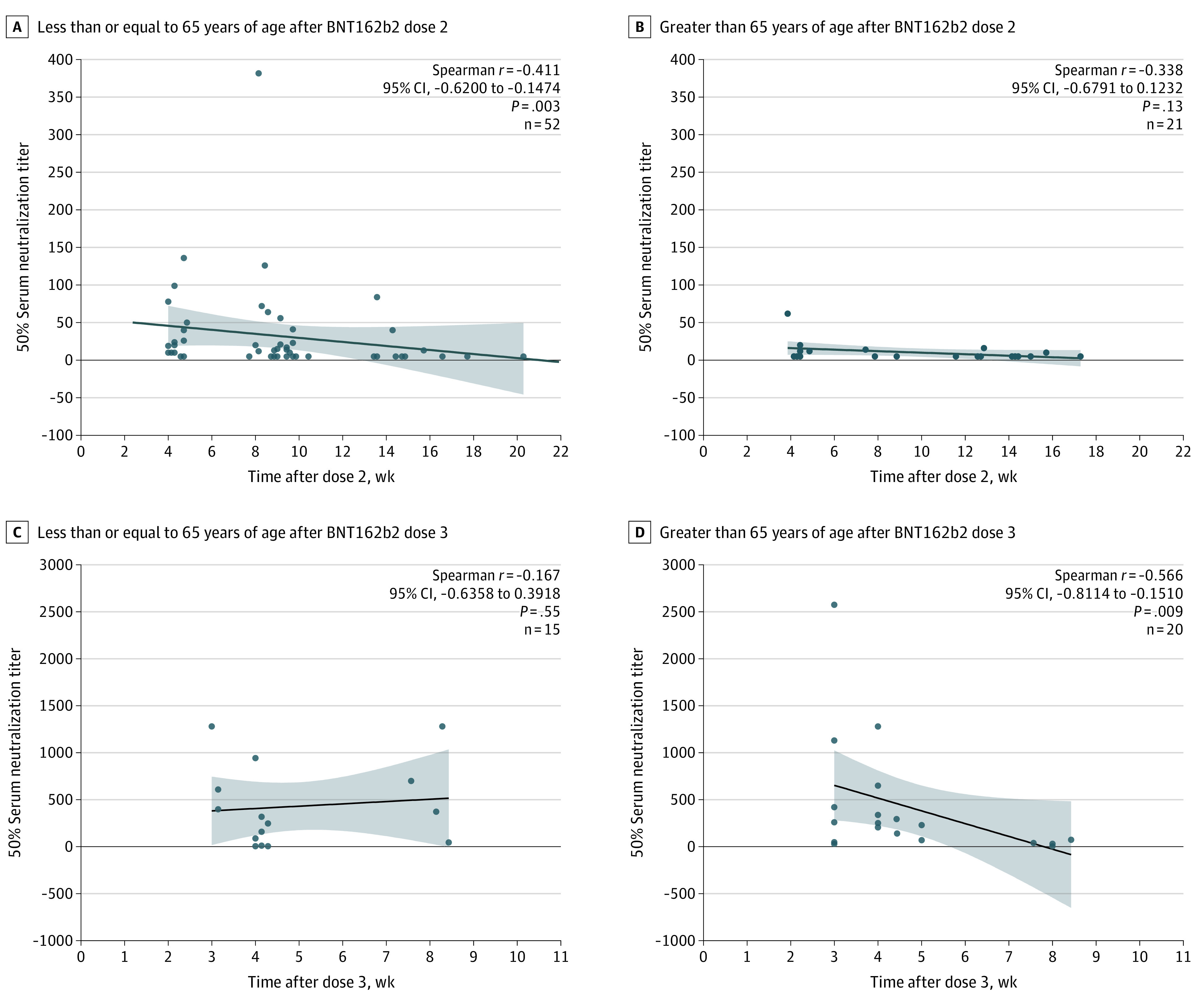
Temporal Omicron Virus Neutralization Titers Following 2 or 3 BNT162b2 Doses in Different Age Groups Individuals who received the primary 2-dose BNT162b2 vaccination series (n = 73; sampling between peak responses at 4 weeks and the last time point, 16 weeks, after dose 2) or 3 BNT162b2 doses (n = 35; sampling between peak responses at 3 weeks and the last time point, 8 weeks) were categorized according to 2 different age groups: less than or equal to 65 years of age and greater than 65 years of age. Omicron-specific neutralization titers for each age group were stratified according to time after vaccine dose. The dot plots represent 50% serum neutralization titers relative to day of sample collection after the second or third BNT162b2 dose for each age group. Each data point presents an individual sample; the black lines represent the linear regression curve and the shaded areas the 95% CI. The association between the 2 continuous variables were assessed using the Spearman correlation test.

The study cohort included 128 vaccinated individuals who received either 2 doses of BNT162b2 (n = 73; median [IQR] age: 51 [37-68] years; 32 [43.8%] male individuals) or 3 doses of BNT162b2 (n = 55; median [IQR] age: 70 [58-79] years; 21 [38.9%] male individuals) administered 4 to 9 months after dose 2 (median [IQR]: 6.9 [6.2-7.5] months). The infected-then-vaccinated individuals (n = 7) were male individuals between 47 and 65 years of age (median [IQR]: 57 [53-62] years). Four weeks after BNT162b2 dose 2, neutralization geometric mean titers (GMTs) against the Omicron variant measured 14-fold lower compared with GMTs against D614G (*P* < .001) (Figure 1A). Relative to D614G and the Delta variant, the proportion of detectable Omicron-specific neutralizing antibody responses declined rapidly from 76.2% (16 of 21 individuals) at week 4 to 53.3% (16 of 30 individuals) at weeks 8 to 10 and 18.9% (3 of 16 individuals) at weeks 12 to 14 (Figure 1A). After BNT162b2 dose 3, GMTs against the Omicron variant increased 20.6-fold at week 3 and 7.7-fold at week 4 compared with GMTs after dose 2 at week 4 (*P* < .001). A third BNT162b2 dose elicited detectable neutralizing antibody responses in the majority of individuals for at least 8 weeks; however, between week 3 and week 8, neutralizing antibody GMTs declined by 4.9-fold for D614G, 5.6-fold for Delta, and 5.4-fold for Omicron. When stratified according to age, GMTs for Omicron-specific neutralizing antibody responses differed significantly between individuals aged less than or equal to 65 years and greater than 65 years after dose 2 (*P* = .02), but not after dose 3 (Figure 1B). However, for the 5 individuals aged greater than 65 years studied at week 8 after dose 3, Omicron-specific GMTs were undetectable for 2 individuals and low (GMT: 30-79) for 3 individuals (Figure 2). Overall, neither age nor interval between doses 2 and 3 were associated with neutralization titers measured between 2 and 4 weeks after dose 3. Similar to a third BNT162b2 dose after the primary 2-dose series, vaccination following a prior infection significantly increased Omicron-specific GMTs (*P* = .02; Figure 1C).

## Discussion

SARS-CoV-2 neutralizing antibodies are correlated with protection against infection and disease.^6^ Our study found a rapid decline in Omicron-specific serum neutralizing antibody titers only a few weeks after the second and third doses of BNT162b2. A limitation of our study is that its cross-sectional design precludes evaluation of antibody decrease rates on an individual level. Nevertheless, the observed decrease in population neutralizing antibody titers corresponds to the decrease in vaccine efficacy against polymerase chain reaction–confirmed Omicron infection in Denmark and symptomatic Omicron infection in the United Kingdom.^3,4^ Taken together, vaccine-induced protective antibody responses following a second and third dose of BNT162b2 are transient and additional booster doses may be necessary, particularly in older people; however, conserved T-cell immunity and nonneutralizing antibodies may still provide protection against hospitalization and death.

## Notes

Supplement. **eAppendix.** Materials and Methods
eReferences
