# Supplementary material for: Neutralizing Antibodies Against the SARS-CoV-2 Omicron Variant (BA.1) 1 to 18 Weeks After the Second and Third Doses of the BNT162b2 mRNA Vaccine
Source: JAMA Netw Open. 2022 May 13;5(5):e2212073. doi: 10.1001/jamanetworkopen.2022.12073 (PMC9107029; doi:10.1001/jamanetworkopen.2022.12073)
Supplement: Supplement. — eAppendix. Materials and Methods eReferences [file jamanetwopen-e2212073-s001.pdf]

Supplemental Online Content

Lassaunière R, Polacek C, Frische A, et al. Neutralizing antibodies against the SARS-CoV-2 Omicron variant (BA.1) 1 to 18 weeks after the second and third doses of the BNT162b2 mRNA vaccine. *JAMA Netw Open.* 2022;5(5):e2212073. doi:10.1001/jamanetworkopen.2022.12073

eAppendix. Materials and Methods

eReferences

This supplemental material has been provided by the authors to give readers additional information about their work.

© 2022 Lassaunière R et al. *JAMA Network Open.*

MATERIALS AND METHODS

Cohorts

Primary 2-dose mRNA vaccination series. The cross-sectional Danish cohort of individuals (n=73) who received two doses of Comirnaty (BNT162b2; Pfizer/BioNTech; the predominant SARS-CoV-2 vaccine used in Denmark [85.8%]) were between 20 and 91 years of age (median: 51 years; interquartile range (IQR): 37−68 years); 32/73 (43.8%) were males. The age distribution was as follows: 20−29 years (n=9); 30−39 years (n=13); 40−49 years (n=10); 50−59 years (n=13); 60−69 years (n=11); 70−79 years (n=10); and ≥80 years (n=7). Only individuals who tested positive for SARS-CoV-2 spike receptor binding domain (RBD) antibodies in the Wantai Total Ab ELISA (Beijing Wantai Biological Pharmacy, Beijing, China) were included. Individuals received their first dose between 18. January 2021 and 15. May 2021. Serum samples were collected ≤16 weeks after the second dose between 17. February 2021 and 8. July 2021.

Third dose mRNA vaccination. A separate cross-sectional Danish cohort included individuals (n=54) who received a third dose of Comirnaty (BNT162b2) between 11. September 2021 and 28. October 2021, at a median interval of 6.5 months (range: 4−9 months) after the second dose. Individuals were between 22 and 94 years of age (median: 70 years; IQR: 58−79 years); 21/54 (38.9%) were males. This cohort comprise older individuals due to the initial prioritizing of a third vaccine dose to protect those ≥65 years of age in Denmark. The age distribution in this group were as follows: 20−29 years (n=2); 30−39 years (n=2); 40−49 years (n=2); 50−59 years (n=10); 60−69 years (n=14); 70−79 years (n=12); and ≥80 years (n=12). Only individuals who tested positive for SARS-CoV-2 spike RBD antibodies in the Wantai Total Ab ELISA (Beijing Wantai Biological Pharmacy) were included. Serum samples were collected 1 to 8 weeks after the third dose between 21. September 2021 and 10. November 2021.

Previously infected individuals. Seven male convalescent plasma donors were tested for virus neutralization after a SARS-CoV-2 infection and subsequent vaccination. The donors were healthy and between 47 and 65 years of age (median: 57 years; IQR: 53−62 years). Post-infection samples were taken 46−186 days after a PCR positive test (median: 65 days) and between 19. May 2020 and 9. February 2021. Given this timeframe, individuals became infected with ancestral SARS-CoV-2 strains before variants of concern became dominant in Denmark. For 6 of the 7 vaccinated donors, samples were taken within 5 weeks after vaccination; sampling time since vaccination for one donor could not be determined. Post-vaccination samples were taken between 8. June 2021 and 6. July 2021. The exact vaccine type and number of doses are unavailable. However, in Denmark, the predominant vaccines used are Comirnaty (BNT162b2) at 85.8% and SpikeVax (mRNA-1273, Moderna) at 13.2% given as two doses in those with and without previous infection. Denmark suspended the use of recombinant adenoviral vectored vaccines from AstraZeneca and Janssen on 14. April 2021 and 3. May 2021, respectively. Thus, the donors very likely received an mRNA vaccine. Of note, a single mRNA dose after infection already significantly increases neutralizing antibody titers after 2−3 weeks, these high titers are not significantly increased by a second mRNA vaccine dose[1,2].

Virus isolation

In a 24-well plate, 4×10^4 Vero E6 cells were seeded per well and cultured overnight in culture medium (Dulbecco's Modified Eagle Medium [DMEM], 10% fetal calf serum and 1% Penicillin/Streptomycin) at 37°C, 5% CO_2. Prior to inoculation, the cell culture media was removed, the monolayer washed once with 1 mL phosphate buffered saline (PBS) and covered with 250 µL infection media (DMEM and 1% Penicillin/Streptomycin). Throat swabs submerged in PBS were used for the primary isolation of the SARS-CoV-2 Omicron BA.1 variant of concern and other SARS-CoV-2 strains. A

© 2022 Lassaunière R et al. *JAMA Network Open.*

150 µL of the sample was added dropwise to an 80−90% confluent Vero E6 cell monolayer. The cells were incubated with the inoculum for 1 hour at 37°C, 5% CO_2, followed by the addition of 1 mL growth media (DMEM, 1% Penicillin/Streptomycin, 1.5 µg/mL Amphotericin B, 10% fetal calf serum). The cell culture supernatant was harvested 72-96 hours post-inoculation, and stored at -80°C. All cell culture reagents were from Gibco, ThermoFisher Scientific, Waltham, Massachusetts, USA.

Virus propagation

To expand the virus stocks, 1.5 × 10^6 Vero E6 cells were seeded in a 75-cm^2 flask in culture medium and incubated overnight at 37°C, 5% CO_2. The cell monolayer was washed once with 5 mL PBS and inoculated with 20-100 µL Passage-1 virus diluted in 2 mL DMEM. After a 1 hour incubation with the inoculum at 37°C, 5% CO_2, 10 mL virus growth media (DMEM with 5% fetal calf serum, 1% Penicillin/Streptomycin, and 10 mM HEPES buffer) was added and incubated at 37°C, 5% CO_2. The virus was harvested 96 hours later. The passage-2 virus stock was clarified by centrifugation at 300 × g for 5 minutes and the supernatant stored in single use aliquots at -80°C. All experiments were performed with the passage-2 stock.

Virus titration

In a 96 well tissue culture plate, 1 × 10^4 Vero E6 cells were seeded per well and incubated overnight at 37°C, 5% CO_2. Prior to inoculation, the cell monolayer was washed once with 200 µL PBS. A 100 µL of SARS-CoV-2 virus serially diluted in virus diluent (DMEM, 1% Penicillin/Streptomycin, 2% bovine serum albumin, and 10 mM HEPES buffer) was added to the cells and incubated at 37°C, 5% CO_2. Infection of wells was confirmed using an anti-SARS-CoV-2 nucleocapsid protein ELISA (described below) at 96 hours post-inoculation where virus growth plateaued. Virus stock titers were calculated by the Reed and Muench method and expressed as the 50% tissue culture infectious dose per mL ($TCID_{50}$/mL). The passage-2 virus stocks, stored in single use aliquots at -80°C, were titrated in triplicate. Each replicate titration assay tested quadruplicate serial dilutions of each virus. The average titer of the triplicate measurements were used for inoculum determinations.

Virus microneutralization test

A 2-fold serial dilution of heat-inactivated serum/plasma samples were mixed with 300 × $TCID_{50}$ SARS-CoV-2 virus and the solution incubated for 1 hour at 37 °C, 5% CO_2. The diluted serum/plasma with virus was subsequently added to Vero E6 cells (kindly provided by Bjoern Meyer, Institut Pasteur, Paris, France) in a 96 well tissue culture plate, seeded with 10^4 cells per well the day prior, and incubated at 37 °C, 5% CO_2. The following day, the inhibition of virus infection was measured in a standard ELISA targeting the SARS-CoV-2 nucleocapsid protein. Culture medium was removed from the infected Vero E6 cell monolayers and the cells washed twice with 100 µL PBS. The cells were fixed with cold 80% (v/v) acetone in PBS for 10 minutes. Following three wash steps with wash buffer (PBS containing 1% (v/v) Triton-X100) for 30 seconds, a 100 µL of a SARS-CoV-2 nucleocapsid protein mouse monoclonal antibody clone 7E1B (1:4000 dilution; Cat. # BSM-41414M, Bioss, Woburn, Massachusetts, USA) was added and incubated for 5 minutes on an orbital shaker (300 rpm) at room temperature and subsequently for 1 hour at 37°C. The plates were washed and incubated with 100 µL of a 1:10000 diluted goat anti-mouse IgG (H+L) cross-adsorbed HRP conjugate antibody (Cat. # A16078; Invitrogen, Waltham, Massachusetts, USA) for 5 minutes on an orbital shaker (300 rpm) at room temperature and subsequently for 1 hour at 37°C. The plates were washed five times with wash buffer for 30 seconds, followed by three washes with deionized water. A 100 µL TMB One Substrate (Cat. # 4380, KemEnTec, Denmark) was added and incubated for 15 minutes. The reaction was stopped with 100 µL H_2SO_4 and absorbance read at 450 nm using 620 nm as a reference on a FLUOstar Microplate Reader (BMG LABTECH, Germany).

© 2022 Lassaunière R et al. *JAMA Network Open.*

Included on each microneutralization plate were quadruplicate wells containing cells with 300 × $TCID_{50}$ SARS-CoV-2 virus without serum (virus control) and quadruplicate wells containing cells with virus diluent only (cell control). The neutralization antibody titer was determined for each serum sample as the interpolation of a four-parameter logistic regression curve with the 50% virus level cut-off calculated for each assay plate: [(mean OD of virus control wells) + (mean OD of cell control wells)]/2. The reciprocal serum dilution corresponding to that well is reported as the 50% serum neutralization titer for that sample. The microneutralization assay is validated and has a comparable performance to other live virus neutralization assays established at different European laboratories (laboratory 4 in ref[3]). Virus neutralization was measured for the following SARS-CoV-2 viruses: early pandemic (Phylogenetic Assignment of Named Global Outbreak Lineages (PANGOLIN) lineage B.1) strain SARS-CoV-2/Hu/Denmark/SSI-H1; Delta variant (PANGOLIN lineage B.1.617.2) strain SARS-CoV-2/Hu/Denmark/SSI-H11; and Omicron variant (PANGOLIN lineage B.1.1.529) strain SARS-CoV-2/Hu/Denmark/SSI-H46. Strains from Denmark were isolated at Statens Serum Institut from clinical samples on Vero E6 cells. All virus stocks were deep sequenced to confirm identity, confirm the absence of cell culture-derived mutations, and the presence of lineage-specific mutations in the spike protein.

Biosafety and security

All experiments were performed in a Biosafety Level 3 (BSL3) laboratory according to standard biosecurity and institutional safety procedures. Biological material used in this study are contained in an access-controlled building and BSL3 laboratory with limited access by appropriately trained personnel. Following each experiment, material containing live virus was inactivated with 2% Virkon S followed by autoclaving.

Statistical analyses

Paired numerical measurements were compared using the Wilcoxon matched-pairs signed rank test. Non-paired numerical measurements were compared using the Mann-Whitney U test. The association between continuous variables were assessed using the Spearman correlation analysis. All statistical analyses and graphing were performed in GraphPad Prism version 9.0.2 (161) (GraphPad Prism Software).

eREFERENCES

1. Saadat S, Rikhtegaran Tehrani Z, Logue J, et al. Binding and Neutralization Antibody Titers After a Single Vaccine Dose in Health Care Workers Previously Infected With SARS-CoV-2. JAMA 2021;325:1467-9.
2. Gobbi F, Buonfrate D, Moro L, et al. Antibody Response to the BNT162b2 mRNA COVID-19 Vaccine in Subjects with Prior SARS-CoV-2 Infection. Viruses 2021;13:422.
3. Nguyen D, Simmonds P, Steenhuis M, et al. SARS-CoV-2 neutralising antibody testing in Europe: towards harmonisation of neutralising antibody titres for better use of convalescent plasma and comparability of trial data. Eurosurveillance 2021;26:2100568.

© 2022 Lassaunière R et al. *JAMA Network Open.*
